# Supplementary material for: An unexpected protein interaction promotes drug resistance in leukemia
Source: Nat Commun. 2017 Nov 16;8:1547. doi: 10.1038/s41467-017-01678-y (PMC5691054; doi:10.1038/s41467-017-01678-y)
Supplement: Supplementary file 1 — Supplementary Information [file 41467_2017_1678_MOESM1_ESM.pdf]

Supplemental Figures

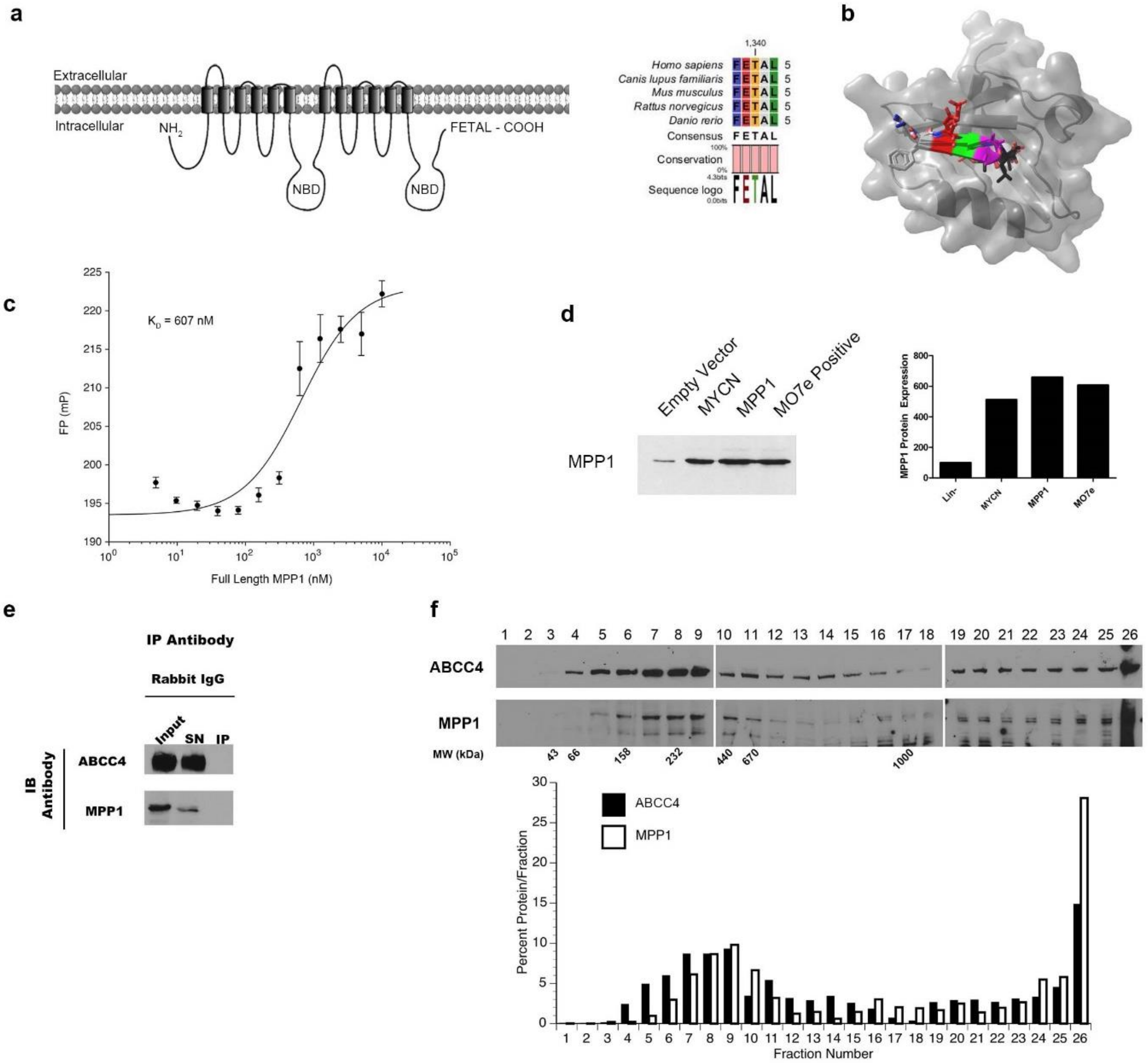

Supplementary Figure 1. ABCC4 has a highly conserved PDZ-motif that binds to purified MPP1 and co-localizes as revealed by gradient fractionation. a) Diagram of ABCC4 (left) showing the highly conserved ABCC4 C-terminal PDZ-binding motif (right). b) Model of MPP1 PDZ-domain interacting with the ETAL-PDZ motif of ABCC4 c) Fluorescent polarization showing binding affinity of FAM-ABCC4 peptide to MPP1. d) Representative immunoblot showing overexpression of MPP1 in mouse hematopoietic progenitor cells transduced with empty vector, MYCN, or MPP1, followed by FACS sorting. MO7e as a positive control. e) Co-immunoprecipitation with non-specific rabbit IgG did not immunoprecipitate ABCC4 or MPP1. f) Glycerol gradient fractionation identifying ABCC4 and MPP1 complex formation. All experiments were performed in triplicate, bars represent s.e.m.

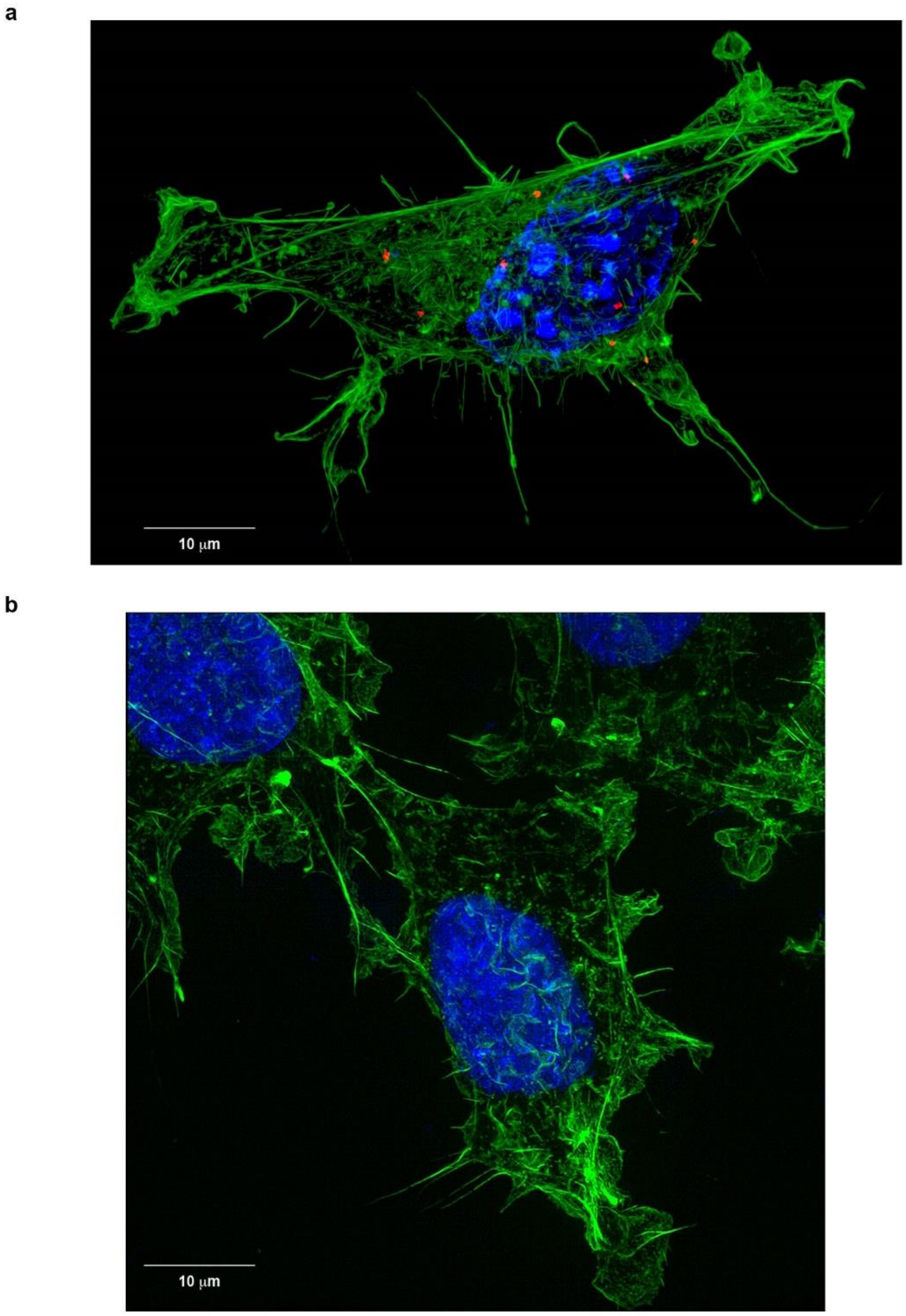

Supplementary Figure 2. Proximity Ligation Assay (PLA) shows MPP1 and ABCC4 only interact when the PDZ-motif of ABCC4 is present. Representative super-resolution maximal intensity projection of PLA products from HEK293 cells transfected with expression vectors for both a) ABCC4 and MPP1 and b) Absence of PLA products in HEK293 cells transfected with an ABCC4 expression vector lacking the PDZ-motif (ABCC4ΔC) and the aforementioned MPP1 expression vector. Colors indicate actin (**green**), dapi (**blue**) and PLA products (**red**) from ABCC4 and MPP1 protein complex.

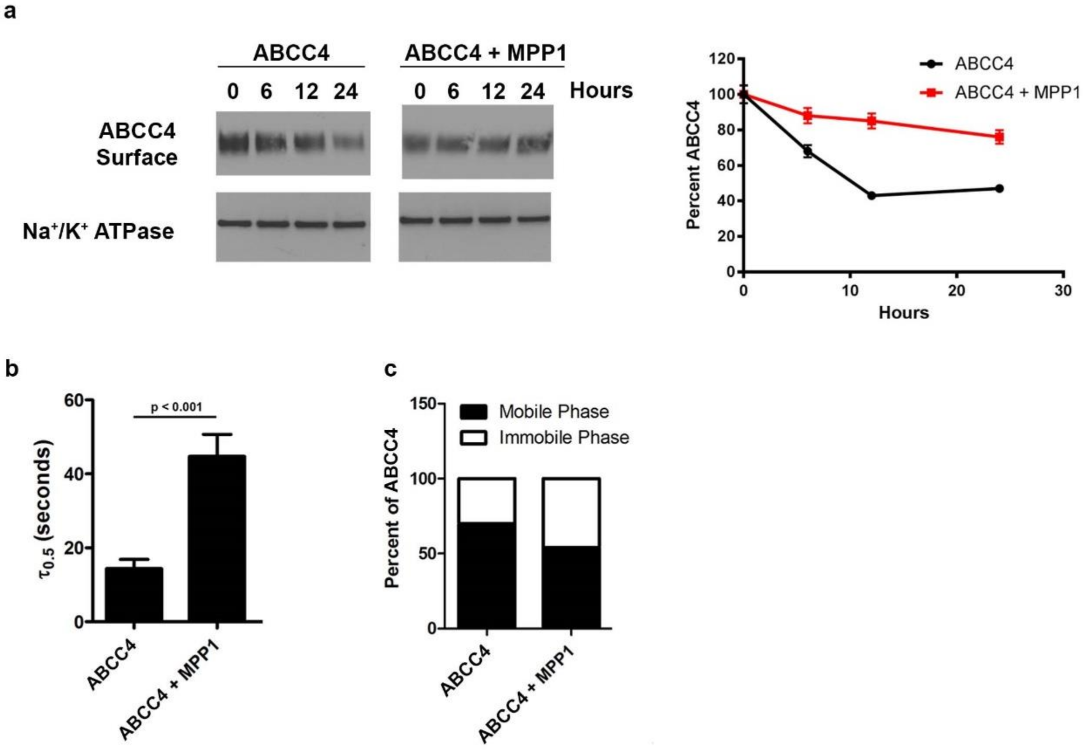

Supplementary Figure 3. Overexpression of MPP1 increases the plasma membrane half-life of ABCC4 and reduces the membrane mobility. a) Cycloheximide (10 µg/ml) was added to cells transfected with either ABCC4 or ABCC4 and MPP1 to estimate ABCC4 turnover. Subsequently the membrane fractions were isolated by surface biotinylation. This turnover experiment indicates that MPP1 increases the ABCC4 half-life at the plasma membrane. b) Fluorescence recovery after photobleaching (FRAP) data was used to calculate half-life to fluorescence recovery ($\tau_{0.5}$). In the presence of MPP1, ABCC4 has increased $\tau_{0.5}$, indicating increased time at the membrane. c) Percentage of ABCC4 in the mobile and immobile phase calculated from FRAP data. In presence of MPP1, ABCC4 has increased immobile fraction. All experiments were performed in triplicate, bars represent s.e.m.

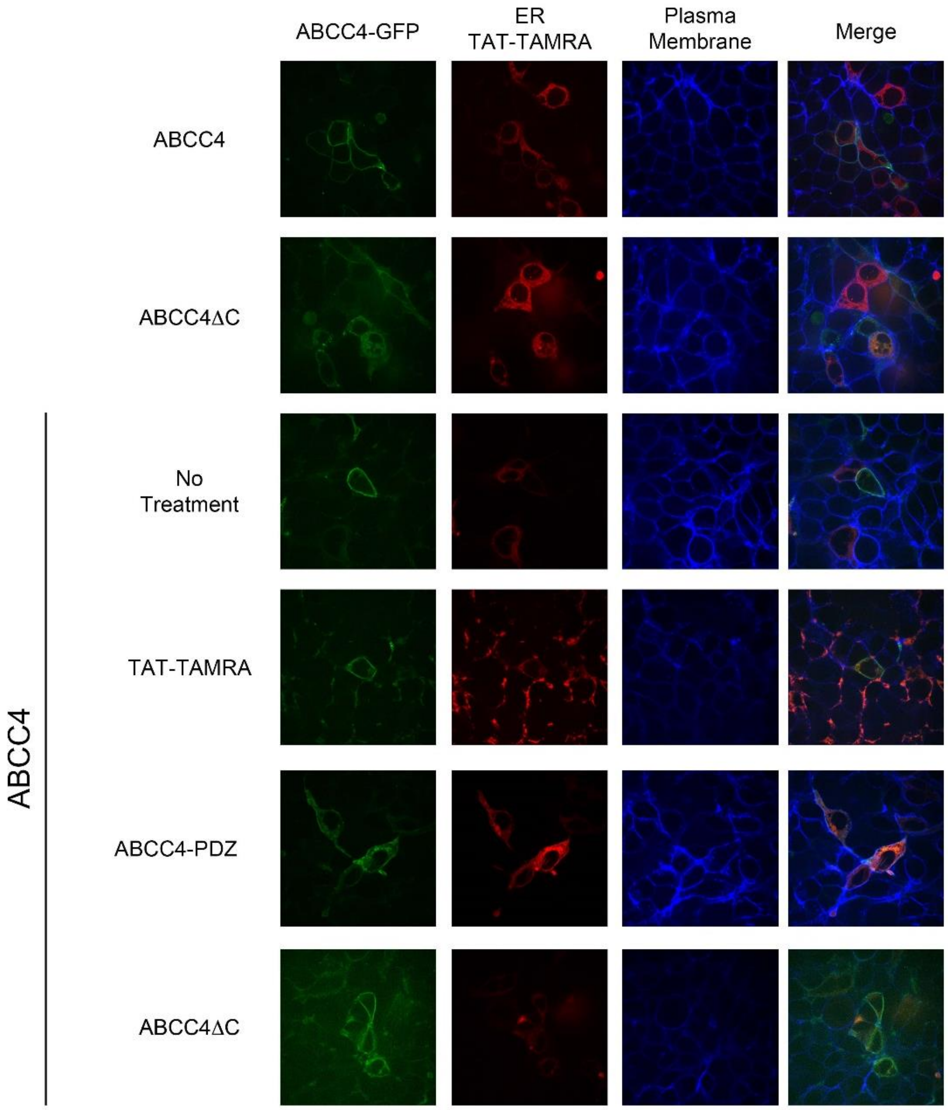

Supplementary Figure 4. Fusion of the cell penetrating TAT-peptide permits intracellular delivery of fluorescent dye (TAMRA) or ABCC4-PDZ motif peptide. Representative images of ABCC4 membrane localization in cells transfected with ABCC4-GFP and MPP1 treated with either a cell-penetrating ABCC4-PDZ motif peptide (10 μM) or lacking the PDZ-motif ABCC4ΔC (10 μM) or the TAT peptide conjugated to TAMRA (10 μM) demonstrating uptake of the peptide.

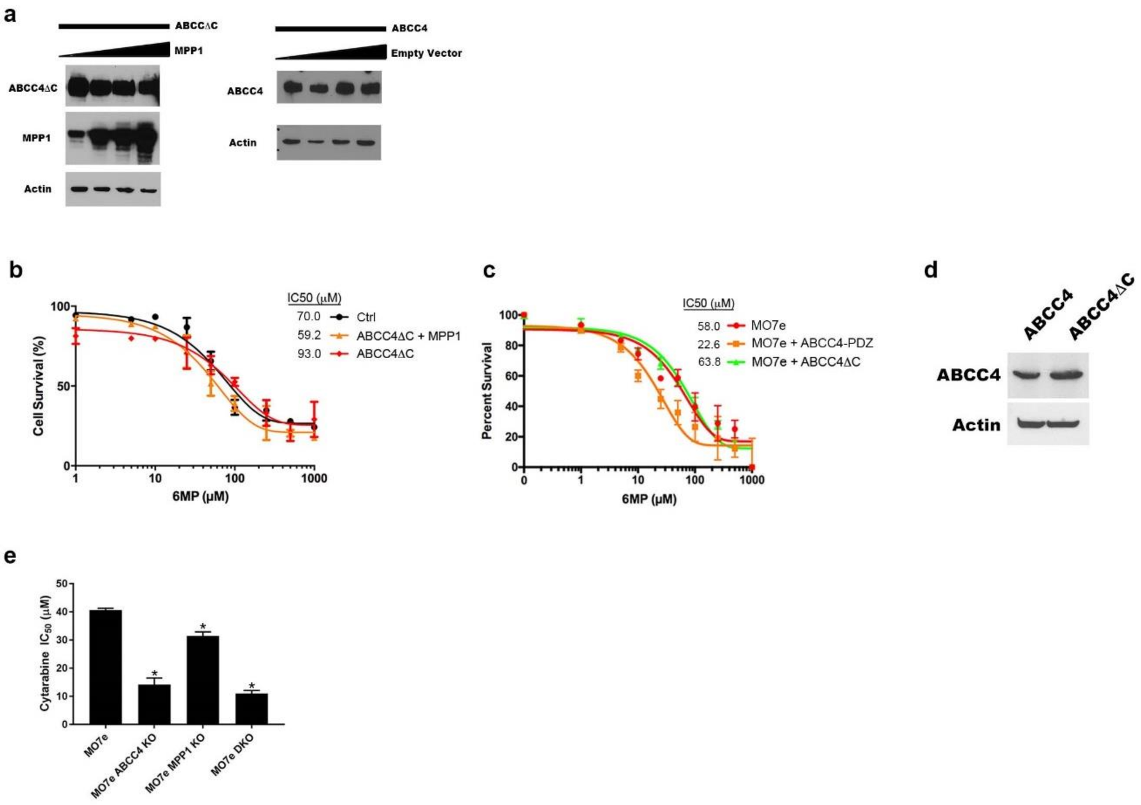

Supplementary Figure 5. MPP1 requires the PDZ-motif of ABCC4 to enhance ABCC4 function. a) HEK293 cells were transfected with a constant amount of an ABCC4ΔC expression vector and various amounts of an MPP1 expression vector. The total amount of DNA was the same in each condition. MPP1 does not alter expression of ABCC4 lacking the PDZ-motif (ABCC4ΔC) (**left**). ABCC4 expression is unchanged by co-transfection of the empty vector plasmid (**right**). b) Survival curve of cells transfected with ABCC4 lacking the PDZ-motif (ABCC4ΔC) is functionally inactive. c) The cell permeable ABCC4-PDZ peptide (10 μM) that blocks the interaction between ABCC4 and MPP1 sensitizes MO7e cells to 6MP. d) Representative image showing equal protein expression in ABCC4 and ABCC4ΔC. All experiments were performed in triplicate, bars represent s.e.m. e) The sensitivity of cells to the non-ABCC4 drug etoposide is not affected by MPP1.

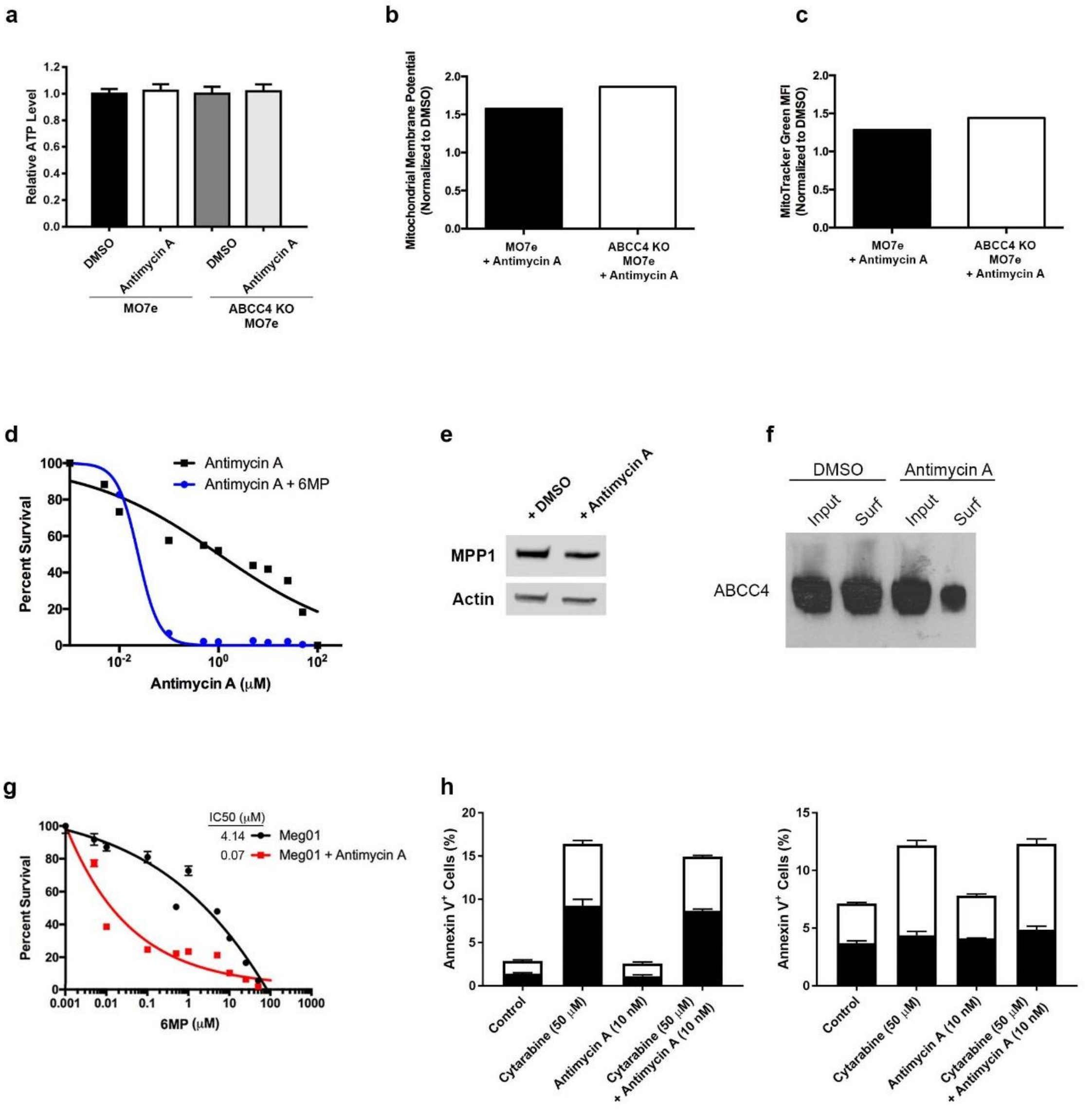

Supplementary Figure 6. Antimycin A does not selectively affect the mitochondrial function of cells lacking ABCC4 or affect the expression of MPP1. a) Equal relative ATP, b) similar mitochondrial membrane potential and c) mitochondrial mass in MO7e and ABCC4 KO MO7e following Antimycin A treatment. d) A Sublethal concentration of 6MP (10 μM) sensitizes MO7e cells to Antimycin A. e) Total protein of MPP1 in Meg01 cells is unchanged following treatement with Antimycin A. f) Surface expression of ABCC4 is altered in Meg01 cells treated with Antimycin A. g) The AML cell line Meg01 was sensitized to 6MP by Antimycin A (10 nM). All experiments were performed in triplicate, bars represent s.e.m. h) Primary AML samples from adult patients show increased

Annexin V^{+} percentage of cells with combination treatment of anitmycin A and cytarabine vs cytarabine alone. Each value was determined from triplicate samples and the error bars represent the s.e.m.

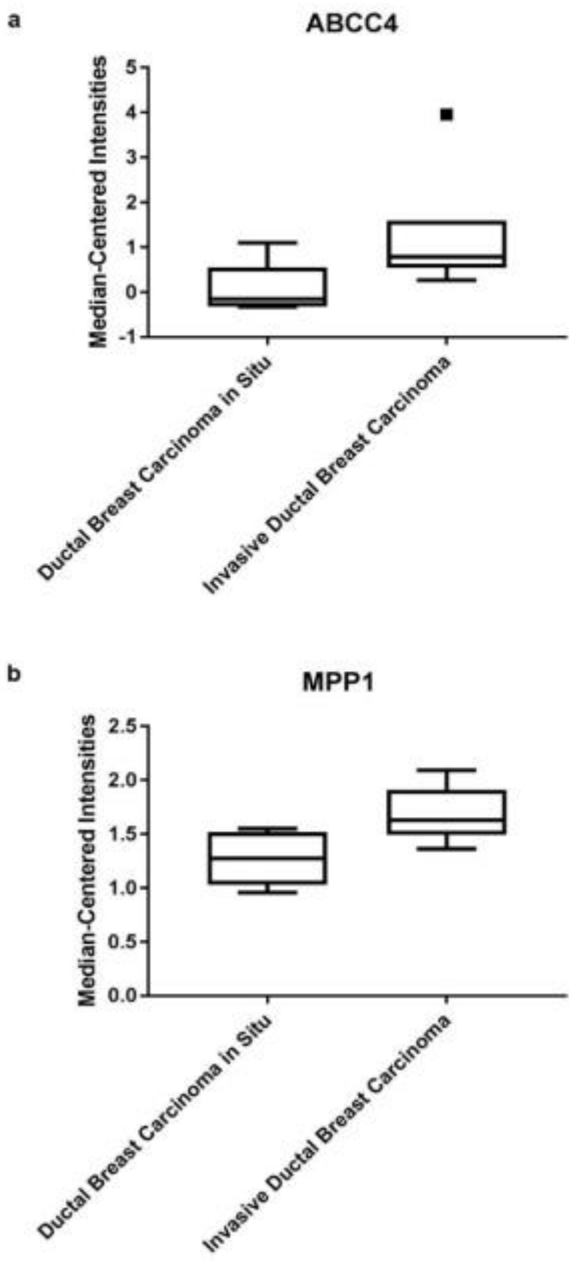

Supplementary Figure 7. More aggressive invasive Breast Carcinomas have higher expression of both ABCC4 and MPP1. a) ABCC4 and b) MPP1 relative expression is increased in invasive ductal breast carcinomas. Analyses were done using the Oncomine database (www.oncomine.org). [37-39]

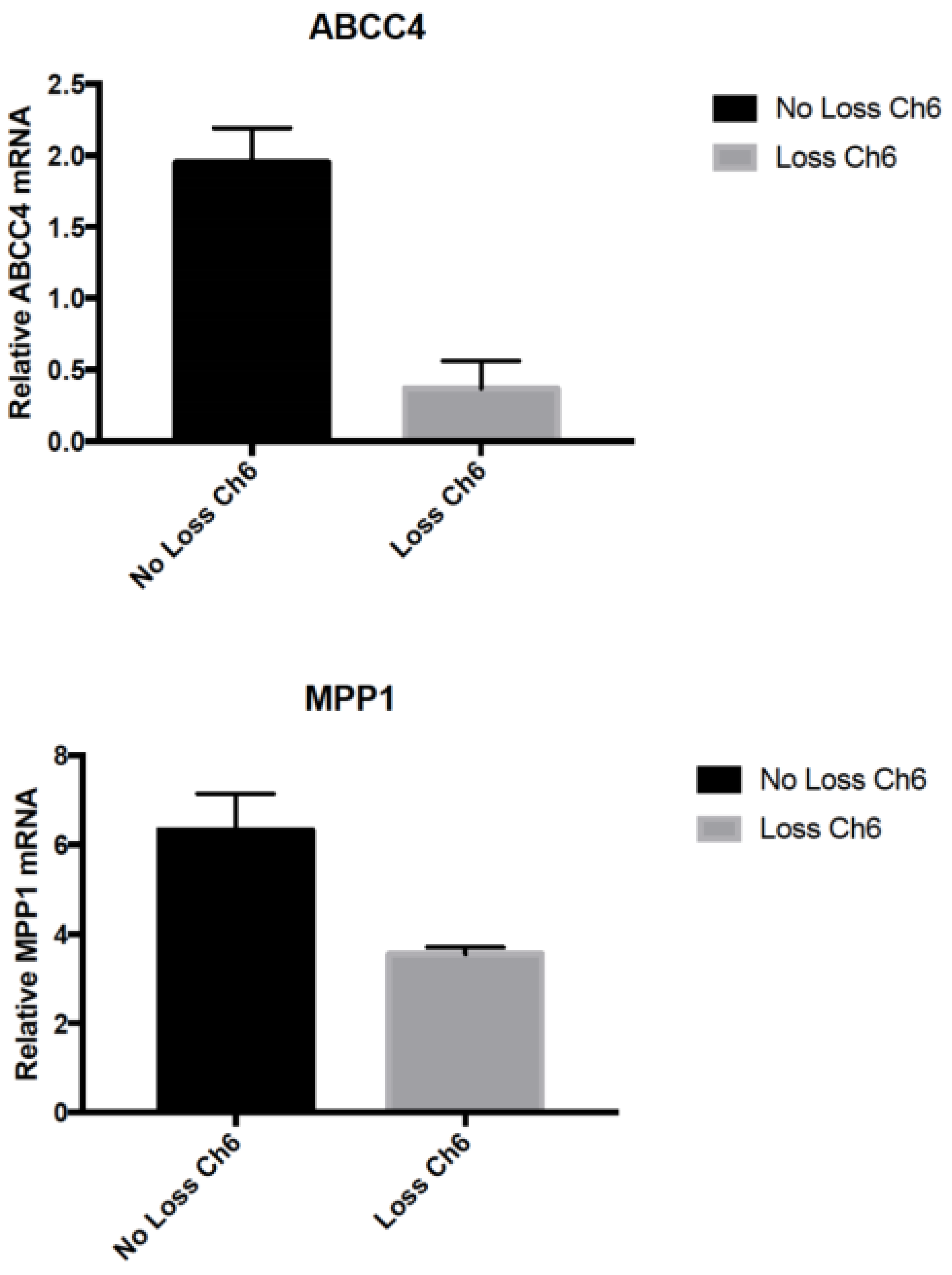

Supplementary Figure 8. Medulloblastomas with no loss of chromosome 6 expresses higher levels of ABCC4 and MPP1. a) ABCC4 and b) MPP1 relative mRNA levels. Values represent the mean. The bar is the standard error of the mean. Analyses were done using the Oncomine database (www.oncomine.org) [37-39]

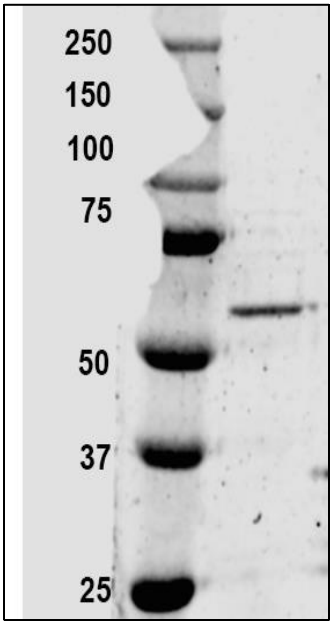

Supplementary Figure 9. Purified His-MPP1.

PAGE gel analysis of purified MPP1.

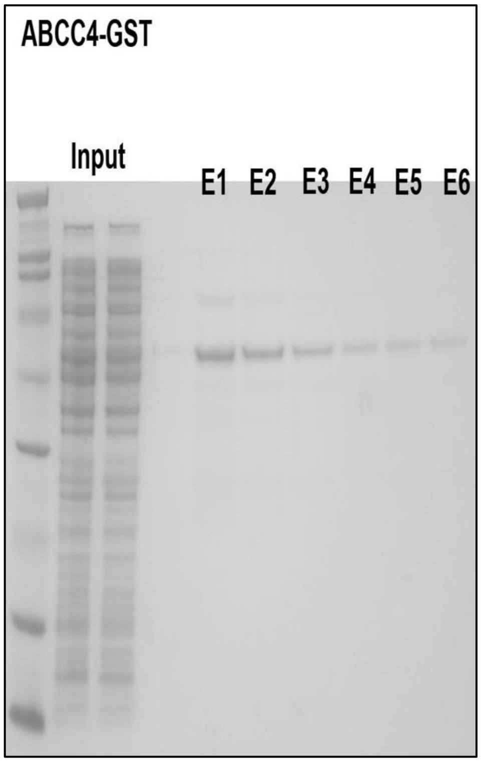

Supplementary Figure 10. PAGE analysis of purified ABCC4-GST.

The fractions E1-3 were discarded and E4-6 combined for our studies.

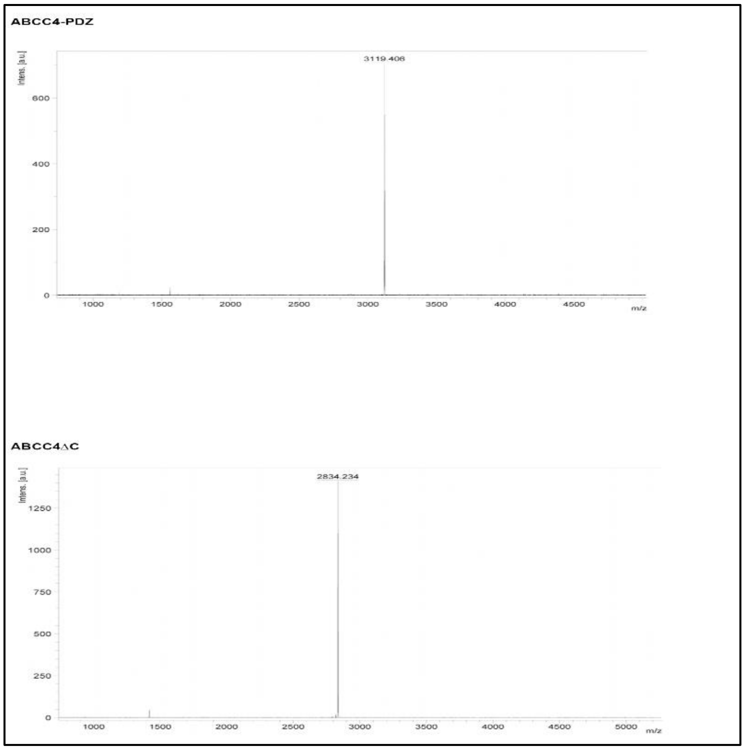

Supplementary Figure 11. Mass-Spec analysis of the ABCC4-PDZ peptide (upper) and the ABCC4∆C (lower) peptide.

Figure 1c

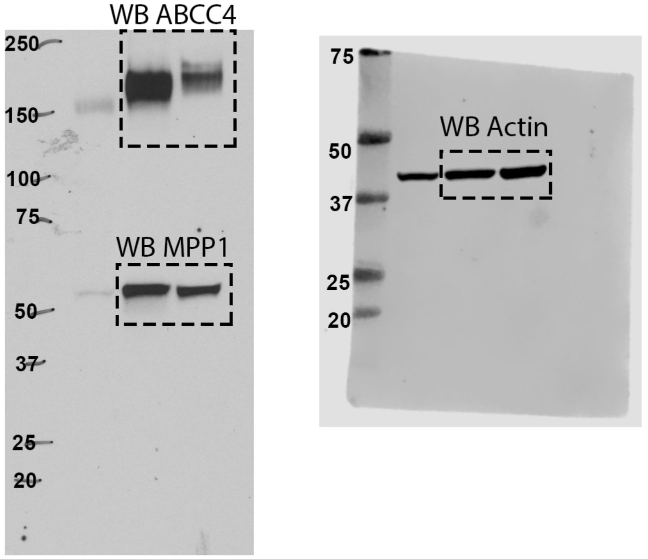

Figure 2b

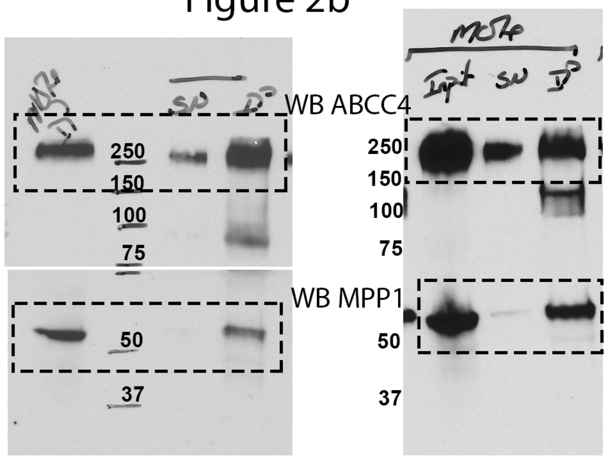

Figure 2c

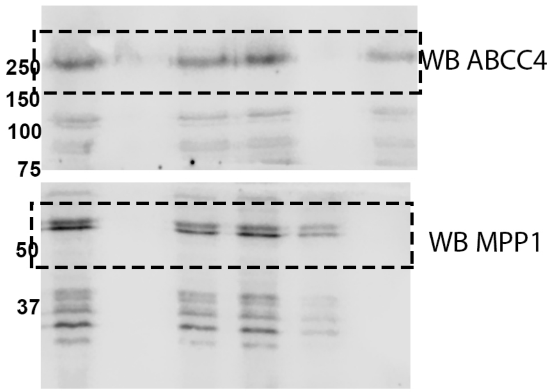

Figure 1f

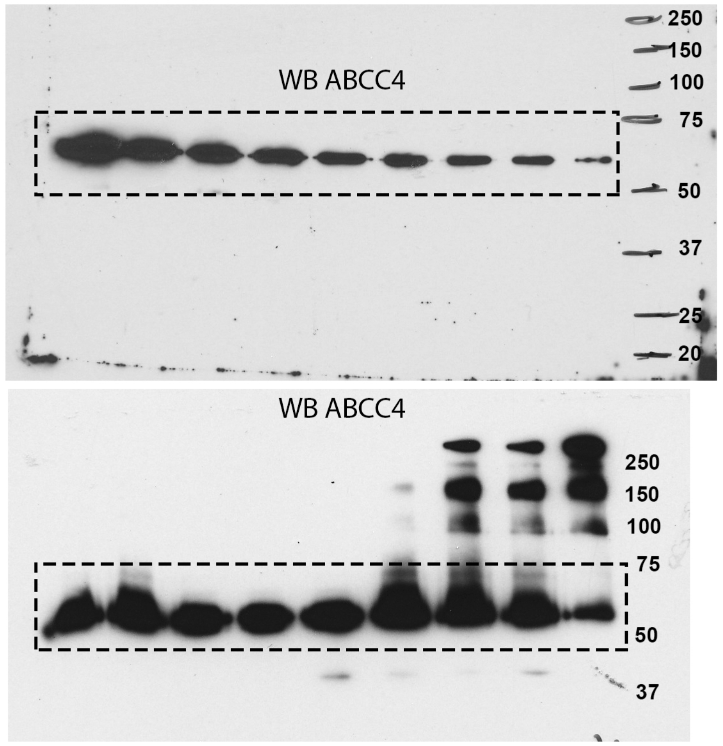

Figure 2e

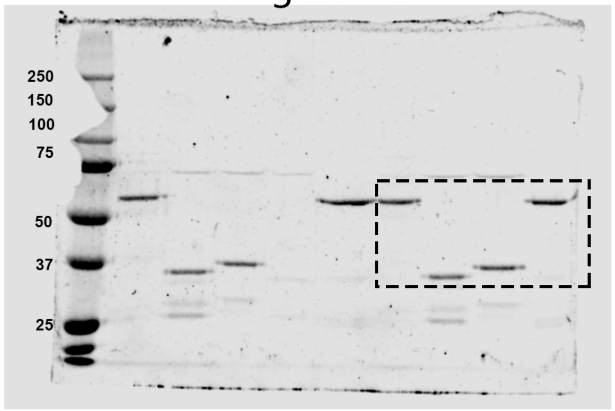

Figure 2f

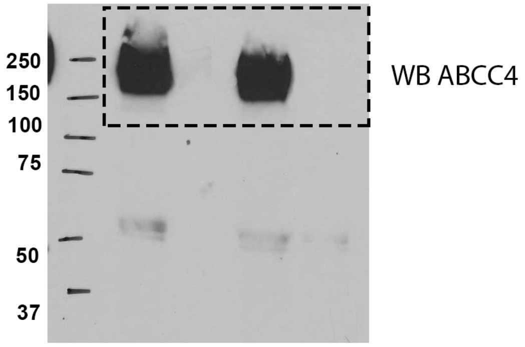

Figure 4a

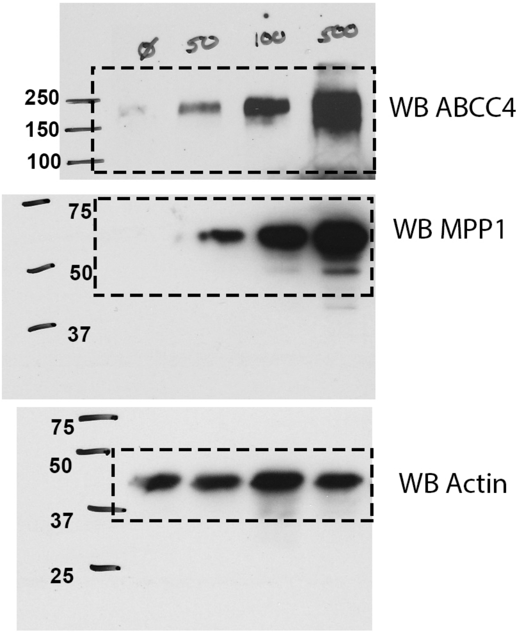

Figure 4d

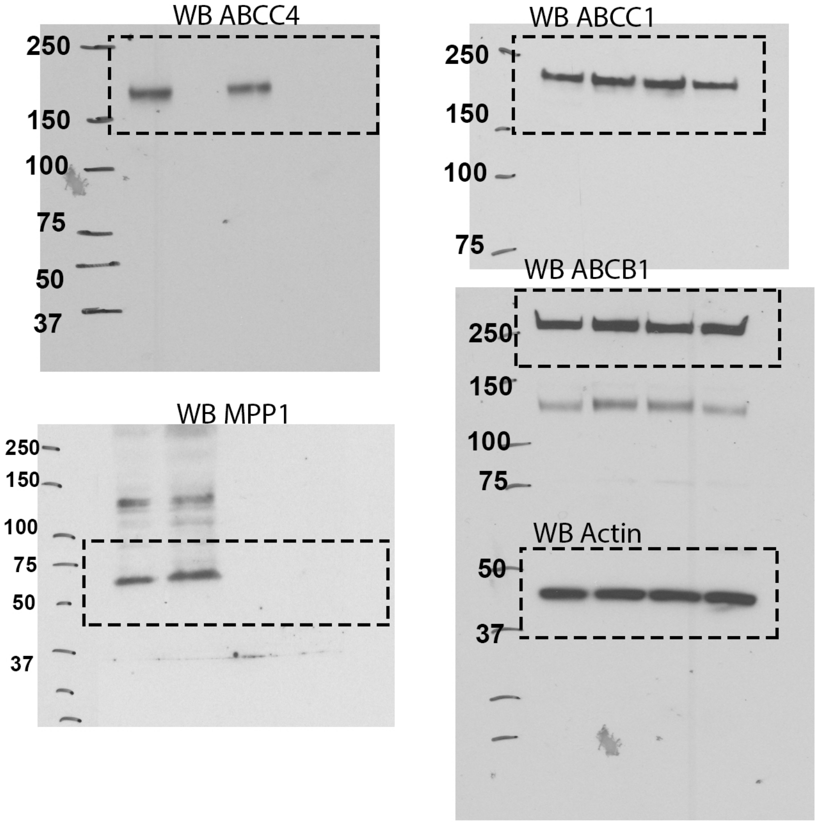

Figure 4g

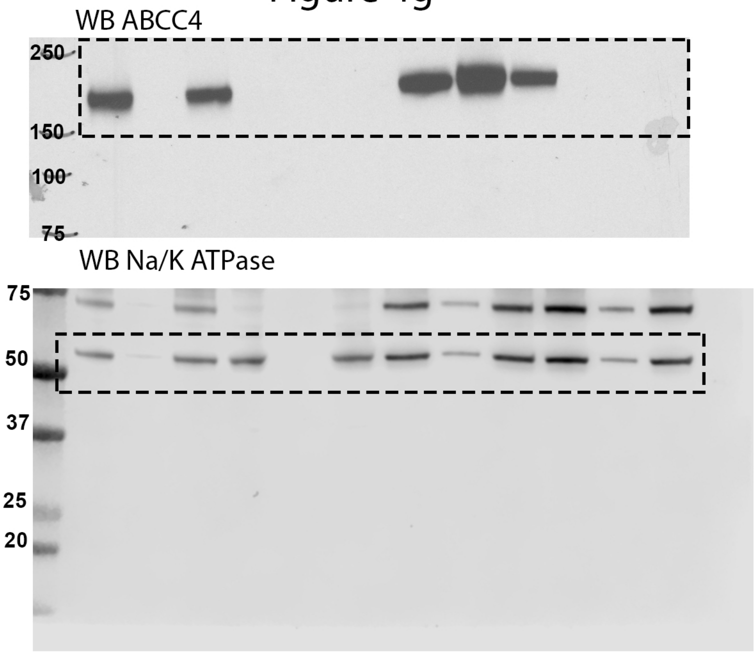

Figure 5d

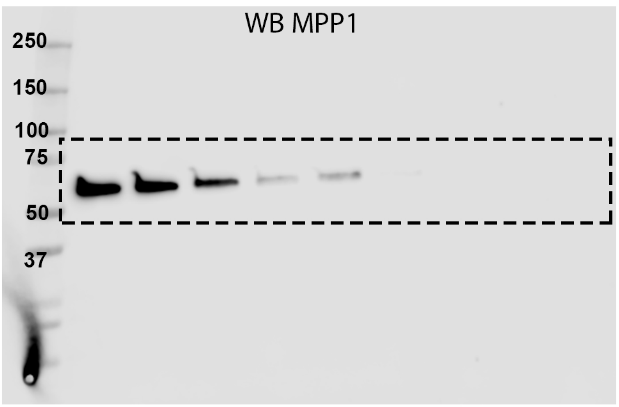

Figure 5j

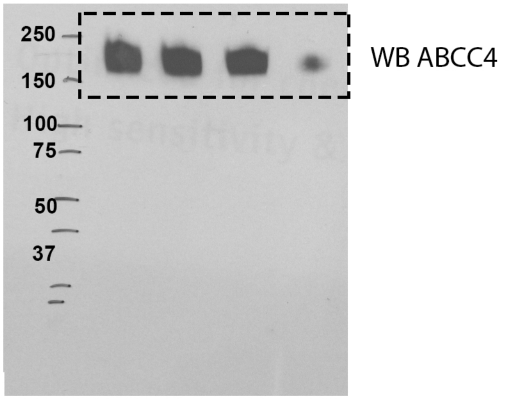

Extended Data Figure 1d

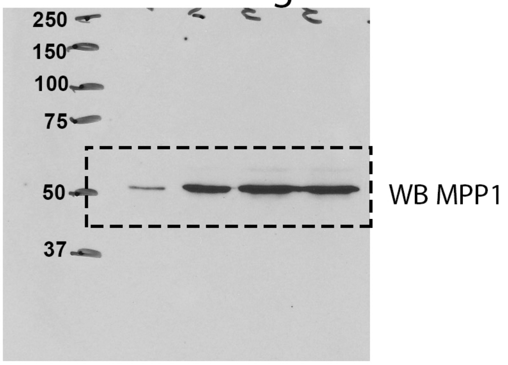

Extended Data Figure 1e

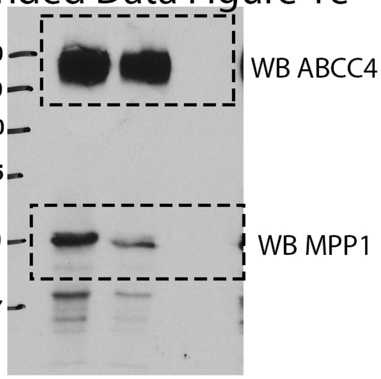

Extended Data Figure 1f

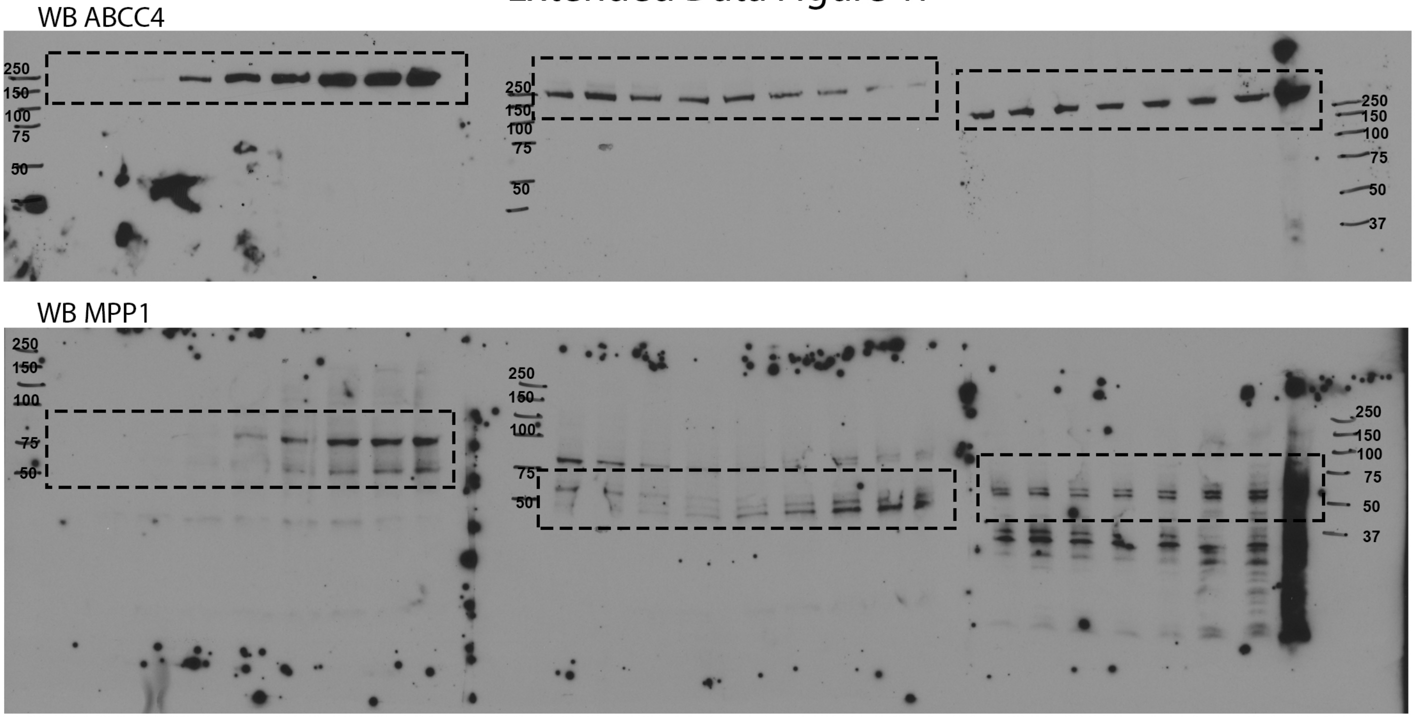

Extended Data Figure 3a

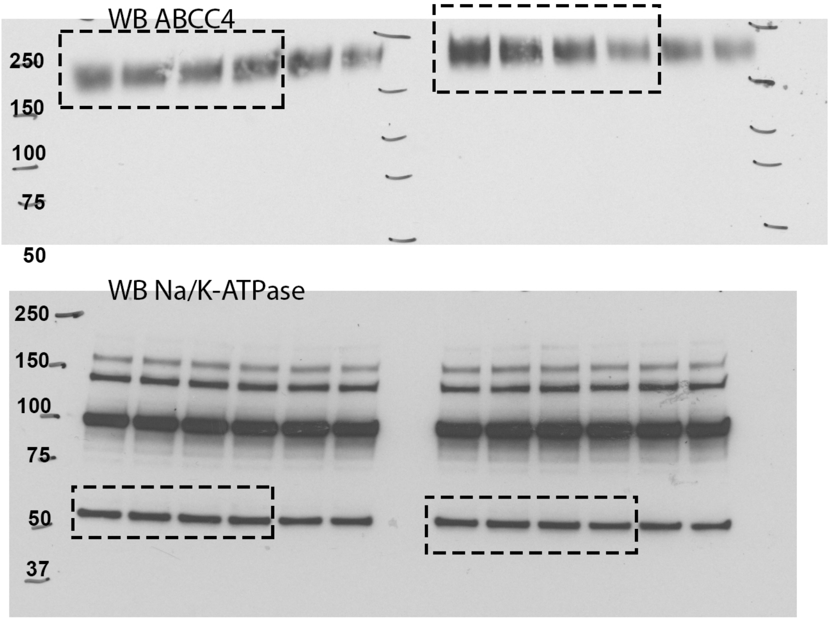

Extended Data Figure 5d

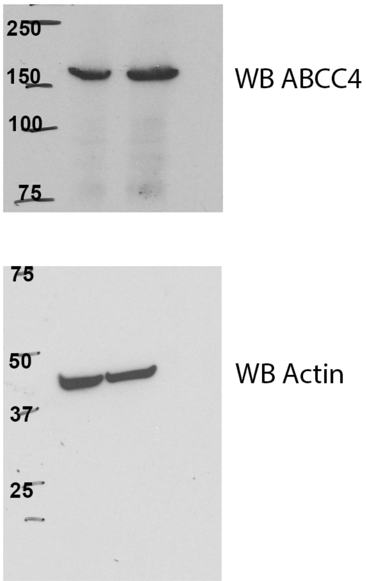

Extended Data Figure 5a

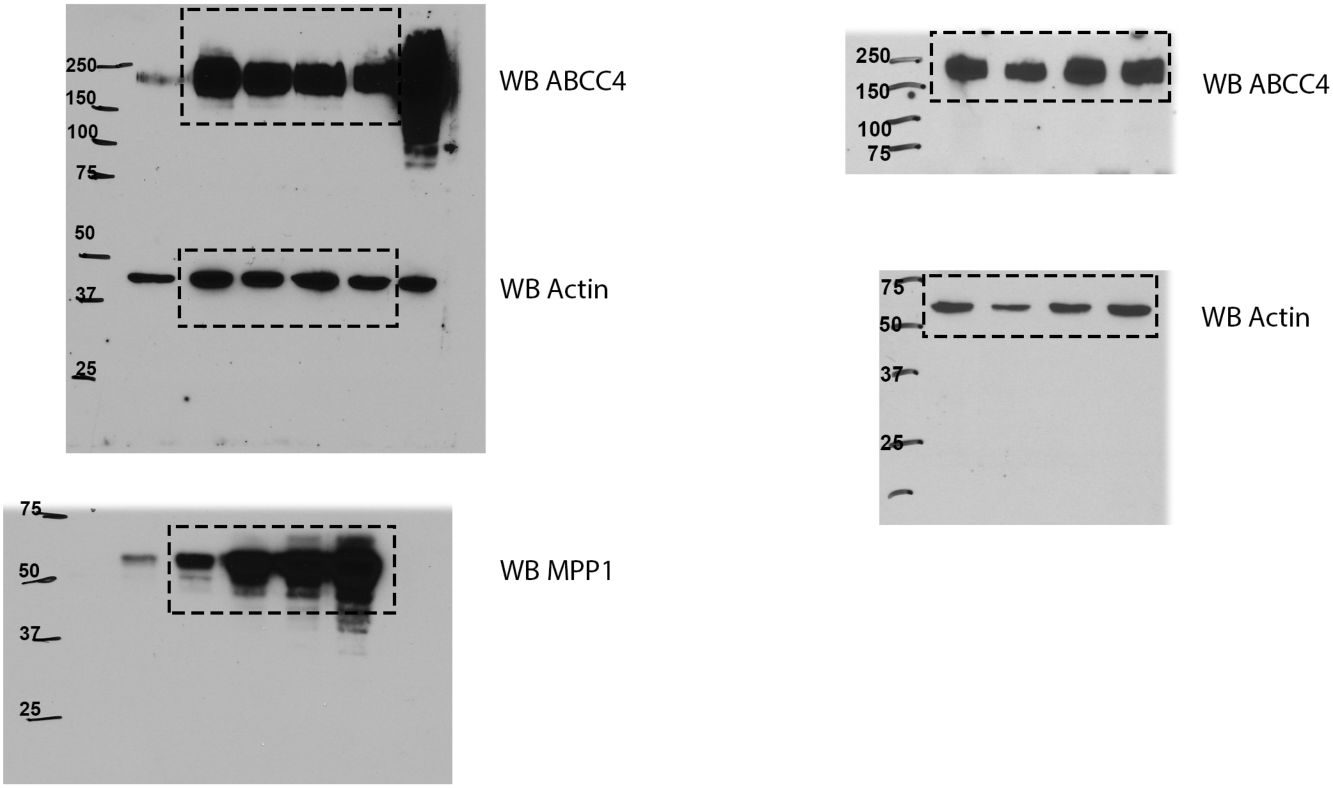

Extended Data Figure 6e

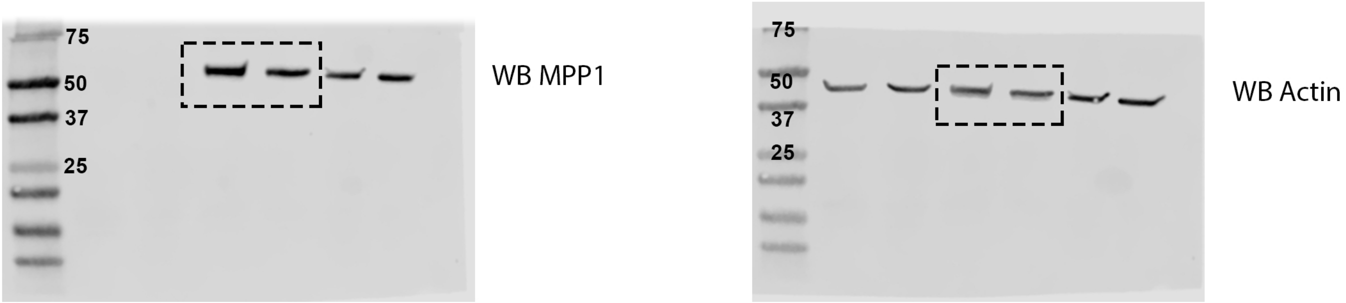

Extended Data Figure 6f

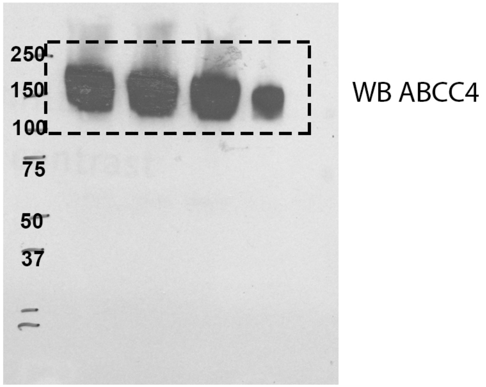

Supplementary Figure 12. Complete immunoblots with cropped areas shown.
